# A novel prognostic signature identifies MFAP4 as a tumor suppressor linking the tumor microenvironment to PI3K/AKT signaling in triple-negative breast cancer

**DOI:** 10.3389/fimmu.2025.1709141

**Published:** 2025-12-10

**Authors:** Xiaoqin Yu, Xiaofen Li, Shiping Luo, Chuangui Song

**Affiliations:** 1 Department of Breast Surgery, Clinical Oncology School of Fujian Medical University, Fujian Cancer Hospital (Fujian Branch of Fudan University Shanghai Cancer Center), Fuzhou, China; 2 Fujian Medical University, Fuzhou, China

**Keywords:** tumor microenvironment, extracellular matrix, MFAP4, PI3K/Akt/mTOR pathway, triple-negative breast cancer

## Abstract

**Background:**

Triple-negative breast cancer (TNBC) remains a clinical challenge due to its aggressiveness and limited therapeutic options. The tumor microenvironment (TME), particularly the extracellular matrix (ECM), is a critical regulator of TNBC progression, yet the key molecular drivers remain largely elusive. This study aimed to identify novel TME-related prognostic biomarkers and elucidate their functional roles in TNBC.

**Method:**

We constructed and validated a multi-gene prognostic model using TNBC datasets from Gene Expression Omnibus (GEO) and The Cancer Genome Atlas (TCGA) databases. The model’s association with TME characteristics was assessed using ESTIMATE algorithm and immune infiltration analyses. The biological functions of the key gene, Microfibril Associated Protein 4 (MFAP4), were investigated *in vitro* via proliferation and migration assays. The underlying mechanism was explored through Western blotting and validated by a functional rescue experiment using a PI3K/AKT/mTOR pathway agonist.

**Results:**

We established a robust 11-gene prognostic model that effectively stratified TNBC patients into high- and low-risk groups with distinct overall and metastasis-free survival. The low-risk group was characterized by an immune-active microenvironment. Notably, MFAP4, an ECM-related gene within the signature, was identified as a key tumor suppressor. MFAP4 expression was significantly downregulated in TNBC tissues and correlated with worse prognosis. Overexpression of MFAP4 markedly suppressed TNBC cell proliferation and migration. Mechanistically, MFAP4 inhibited the phosphorylation of key components of the PI3K/AKT/mTOR pathway. Crucially, pharmacological activation of this pathway with MHY1485 partially rescued the anti-tumor effects induced by MFAP4.

**Conclusion:**

Our study established a TME-centric prognostic signature and, more importantly, identified MFAP4 as a novel tumor suppressor in TNBC. We provide the first evidence that MFAP4 inhibits TNBC malignancy by restraining the PI3K/AKT/mTOR signaling pathway, thereby establishing a critical link between the ECM and intracellular oncogenic signaling. MFAP4 represents a promising prognostic biomarker and a potential therapeutic target for TNBC.

## 1 Introduction

Triple-negative breast cancer (TNBC), characterized by the absence of estrogen receptor, progesterone receptor, and human epidermal growth factor receptor 2 expression, represents a clinically challenging subtype accounting for 15-20% of all breast cancers (1). Distinct from other subtypes, TNBC exhibits a more aggressive phenotype, marked by high invasiveness, early metastasis, and a notably poorer prognosis (2). Despite advances in therapeutic strategies, intrinsic tumor heterogeneity frequently leads to chemoresistance and relapse, posing a formidable hurdle in its clinical management (3). Consequently, there is an urgent and unmet need to identify novel molecular markers that can both refine prognostic stratification and serve as actionable therapeutic targets.

Increasing evidence highlights the tumor microenvironment (TME) as a critical determinant of TNBC’s aggressive nature. Within this complex ecosystem, the extracellular matrix (ECM) has evolved from being viewed as a mere structural scaffold to a dynamic signaling hub that actively regulates tumorigenesis (4). Aberrant remodeling and altered expression of ECM components are intrinsically linked to tumor growth, invasion, and metastatic dissemination (5, 6). Tumors typically promote both collagen over-accumulation in the ECM and upregulation of ECM-modifying enzymes, collectively resulting in pathological elevation of ECM stiffness. This mechanical alteration triggers pro-tumorigenic signaling pathways that facilitate cancer progression (7, 8). Additionally, serving as a cytokine reservoir, the remodeled ECM creates a permissive microenvironment that further supports sustained tumor growth and invasion (4). Nevertheless, the specific contributions of individual ECM proteins to TNBC pathology and their potential utility as prognostic biomarkers remain largely uncharacterized.

Among the diverse constituents of the ECM, Microfibril Associated Protein 4 (MFAP4), a glycoprotein possessing domains critical for cell adhesion and signaling (9, 10), has garnered attention for its context-dependent and often paradoxical roles in oncology. For instance, while MFAP4 acts as a tumor suppressor in lung and hepatocellular carcinoma (11–13), its high expression correlates with poor prognosis in smooth muscle sarcoma and pleomorphic adenomas (14, 15). Although MFAP4 is reportedly downregulated in breast cancer, its precise biological function and clinical significance, particularly within the aggressive TNBC subtype, represent a critical and unaddressed question.

Herein, we sought to bridge this knowledge gap by first developing a TME-informed prognostic signature to systematically identify key ECM-related drivers of TNBC progression. This approach successfully pinpointed MFAP4 as a pivotal candidate. We subsequently elucidated the tumor-suppressive functions of MFAP4 in TNBC and, for the first time, uncovered a mechanistic axis whereby extracellular MFAP4 negatively regulates the intracellular PI3K/AKT/mTOR signaling pathway. Our findings not only establish MFAP4 as a robust prognostic biomarker but also reveal a novel regulatory mechanism, offering a promising therapeutic target for TNBC.

## 2 Materials and methods

### 2.1 Bioinformatic analysis

#### 2.1.1 Data acquisition and preprocessing

Publicly available gene expression datasets and corresponding clinical information were retrieved from the Gene Expression Omnibus (GEO) database (https://www.ncbi.nlm.nih.gov/geo/) and The Cancer Genome Atlas (TCGA) (https://portal.gdc.cancer.gov/). Specifically, GSE5327 and GSE65194 were used for initial gene screening, GSE58812 served as the training cohort for model construction, and GSE53752 was utilized as the validation cohort. TCGA-BRCA (TNBC subset): RNA-sequencing data and clinical information for TNBC patients were extracted from the TCGA-BRCA project.

All GEO datasets were normalized using the limma package in R. FPKM values from TCGA-BRCA were converted to Transcripts Per Million (TPM) for subsequent analyses. A summary of the sample sources, platforms, sizes and other information for the datasets included in this study is provided in **Supplementary Table S1**.

#### 2.1.2 Identification of prognosis-related candidate genes

A Weighted Gene Co-expression Network Analysis (WGCNA) was performed on the GSE5327 dataset to identify gene modules significantly associated with breast cancer progression (*p* ≤ 0.05). Separately, differentially expressed genes (DEGs) between TNBC and adjacent normal tissues were identified in the GSE65194 dataset using the limma package, with thresholds set at an adjusted *p*-value ≤ 0.05 and |log2(Fold Change)| > 2.5. The intersection of genes from the progression-associated modules and the DEGs was taken to form a pool of high-confidence candidate genes for subsequent model construction.

#### 2.1.3 Construction and validation of the prognostic signature

The Least Absolute Shrinkage and Selection Operator (LASSO) Cox regression analysis was employed on the candidate genes in the training cohort (GSE58812) to construct the prognostic signature. The risk score for each patient was calculated using the following formula, derived from the LASSO regression: Risk Score = Σ (βi * Expi), where βi is the regression coefficient and Expi is the expression level of each signature gene. Patients were stratified into high- and low-risk groups based on the median risk score. Survival differences were assessed using Kaplan-Meier analysis. The predictive accuracy of the signature was evaluated by time-dependent Receiver Operating Characteristic (ROC) curve analysis using the timeROC package. The robustness of the signature was independently validated in the GSE53752 cohort.

#### 2.1.4 Functional and immune microenvironment analysis

To explore the biological underpinnings of the signature, DEGs between the high- and low-risk groups were identified. Gene Ontology (GO) and Kyoto Encyclopedia of Genes and Genomes (KEGG) pathway enrichment analyses were performed using the clusterProfiler package (adj.*p* ≤ 0.05). The tumor microenvironment was characterized using the ESTIMATE algorithm to calculate Stromal, Immune, and ESTIMATE scores. The relative abundance of 28 immune cell types was quantified using the single-sample Gene Set Enrichment Analysis (ssGSEA) algorithm within the GSVA package.

### 2.2 Experimental procedures

#### 2.2.1 Cell culture and reagents

The human TNBC cell lines BT-549 and Hs-578T were obtained from the Cell Bank of the Chinese Academy of Sciences (Shanghai, China). Cells were cultured in Dulbecco’s Modified Eagle’s Medium (DMEM, Procell, China) supplemented with 10% fetal bovine serum (FBS, Procell, China) and 1% penicillin-streptomycin (NCM Biotech, China) at 37°C in a humidified incubator with 5% CO_2_. The mTOR agonist, MHY1485, was purchased from MedChemExpress (MCE, the USA) and used at a concentration of 3 μM for 24 h for rescue experiments.

#### 2.2.2 Lentiviral transfection

Lentiviral vectors for MFAP4 overexpression (LV-MFAP4) and the corresponding empty vector control (LV-Vector) were constructed by IGE Biotechnology (Guangzhou, China). Cell suspensions were prepared and inoculated into 6-well plates and cultured until cell confluence reached 20-30%. Transfect the cells with MFAP4 overexpression lentivirus and empty lentivirus respectively according to the instructions. After 72 hours, the transfection efficiency was observed using fluorescence microscopy. Stably transfected cell lines were screened with puromycin (Biosharp, China) for subsequent experiments.

#### 2.2.3 RNA extraction and RT-qPCR

Total RNA was extracted using SPARKeasy Tissue/Cellular RNA Rapid Extraction Kit (Sparkjade, China). cDNA synthesis was performed using All-In-One 5X RT MasterMix with gDNA Removal (abm, Canada). Real-time PCR reactions were performed using Taq Pro Universal SYBR qPCR Master Mix kit (Vazyme, China). GAPDH was used as the housekeeping gene, and the changes in mRNA expression of target genes were calculated by the 2^-ΔΔCT method. Primer sequences are listed in **Supplementary Table S2**.

#### 2.2.4 Western blot analysis

Cells were lysed with RIPA lysate, protease inhibitor, and phosphatase inhibitor (Beyotime, China) to extract proteins, and protein concentration was determined using a BCA kit (Solarbio, China). Equal amounts of protein (30 μg) were separated by SDS-PAGE and transferred to PVDF membranes (Millipore, the USA). Membranes were blocked with 5% non-fat milk for 1 h at room temperature and washed with TBST, followed by incubation of the primary antibody overnight at 4°C. After incubation with HRP-conjugated secondary antibodies (Proteintech, China), protein bands were visualized using an ECL kit (Biosharp, China) on a ChemiDoc Imaging System (Bio-Rad, the USA). A list of all antibodies used is provided in **Supplementary Table S3**.

#### 2.2.5 Cell proliferation assays

##### 2.2.5.1 Cell counting kit-8 assay

Cells were seeded in 96-well plates (2,000 cells/well). At indicated time points (0, 1, 2, 3, 4 days), 10 μL of Cell counting kit-8 (CCK-8) reagent (NCM Biotech, China) was added to each well, and plates were incubated for 2 h. Absorbance at 450 nm was measured using a microplate reader.

##### 2.2.5.2 Colony formation assay

Cells were seeded in 6-well plates (500 cells/well) and cultured for approximately 10–14 days, until visible colonies formed. Colonies were fixed with 4% paraformaldehyde (PFA, Biosharp, China) and stained with 0.1% crystal violet (Biosharp, China). The results were analyzed and counted using Image J software.

##### 2.2.5.3 5-Ethynyl-2’-deoxyuridine assay

Cells were inoculated into 96-well plates according to 5000 cells/well and cultured to the appropriate density. Using 5-Ethynyl-2’-deoxyuridine (EdU) Proliferation Detection Kit (Beyotime, China), cells were fixed, stained and washed according to the instructions, and finally the cells were photographed under an inverted fluorescence microscope, and the proportion of EdU-positive cells was quantified using Image J software.

#### 2.2.6 Cell migration assays

##### 2.2.6.1 Wound healing assay

Cells were seeded in 6-well plates to form a confluent monolayer. A scratch was created using 200 μL pipette tips. Images of the wound were captured at 0 h and 24 h. The wound closure area was quantified using ImageJ software.

##### 2.2.6.2 Transwell migration assay

Cell migration was assessed using 8.0 μm pore size Transwell chambers (Corning, the USA). 2 × 10^4^ cells in serum-free medium were added to the upper chamber, and the lower chamber was filled with DMEM containing 10% FBS. After 16 h, non-migrated cells were removed, and migrated cells on the lower surface were fixed with 4% PFA and stained with 0.1% crystal violet.

### 2.3 Statistical analysis

All *in vitro* experiments were performed with a minimum of three independent biological replicates. Unless otherwise stated, quantitative data are presented as mean ± standard deviation (SD), with individual data points shown in the graphs. Statistical analyses were performed using GraphPad Prism 10 (GraphPad Software, La Jolla, CA). Differences between two groups were compared using a two-tailed Student’s t-test. For comparisons among multiple groups, one-way analysis of variance (ANOVA) followed by Tukey’s *post hoc* test was used. Survival curves were generated using the Kaplan-Meier method and compared with the log-rank test. A *p*-value < 0.05 was considered statistically significant. Significance levels were denoted as *p < 0.05, **p < 0.01, ***p < 0.001, and ****p < 0.0001.

## 3 Results

### 3.1 A TME-associated gene signature is identified as a driver of TNBC progression

**Figure 1:**
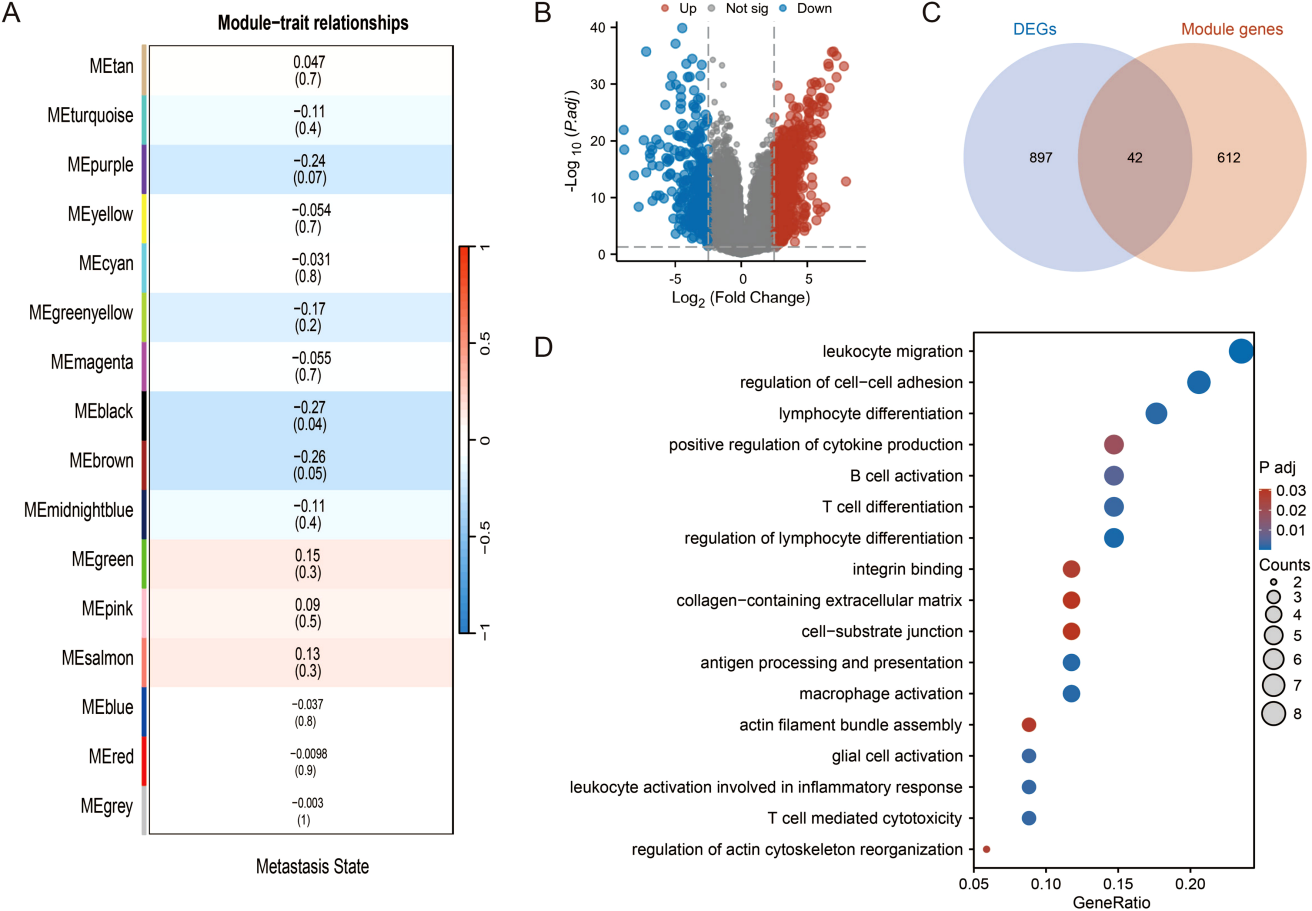
Identification of a TME-associated gene set driving TNBC progression. **(A)** WGCNA identifying gene modules correlated with metastasis status in the GSE5327 dataset. The heatmap depicts the correlation coefficient (and *p*-value) between each gene module (rows) and the metastasis status (column). **(B)** Volcano plot of DEGs between TNBC and normal tissues in the GSE65194 dataset. Red and blue dots represent up- and down-regulated genes, respectively. Genes with an adjusted *p*-value ≤ 0.05 and |log2(Fold Change)| >2.5 are considered significant. **(C)** Venn diagram showing the intersection of WGCNA module genes (MEblanck and MEbrown) and DEGs, yielding 42 candidate genes. **(D)** GO enrichment analysis of the 42 candidate genes. Dot size represents gene count, and color indicates adjusted *p*-value.

To identify key drivers of TNBC progression, we first performed a WGCNA on the GSE5327 dataset. This analysis revealed two gene modules (containing 654 genes in total) that were significantly correlated with metastasis status (**Figure 1A**). Concurrently, we identified 939 DEGs between TNBC and normal tissues in the GSE65194 dataset (**Figure 1B**). By intersecting these two gene sets, we obtained a high-confidence pool of 42 candidate genes robustly associated with TNBC progression (**Figure 1C**). To investigate the potential biological functions of this candidate genes, we performed functional enrichment analysis. Intriguingly, these genes were significantly enriched in TME-related biological processes, including “leukocyte activation involved in immune response”, “regulation of cell-cell adhesion”, and “lymphocyte activation involved in immune response” (**Figure 1D**). This critical finding suggests that the TME landscape is a fundamental determinant of TNBC progression and provides a strong rationale for developing a TME-informed prognostic model.

### 3.2 Construction and validation of a robust TME-informed prognostic signature

**Figure 2:**
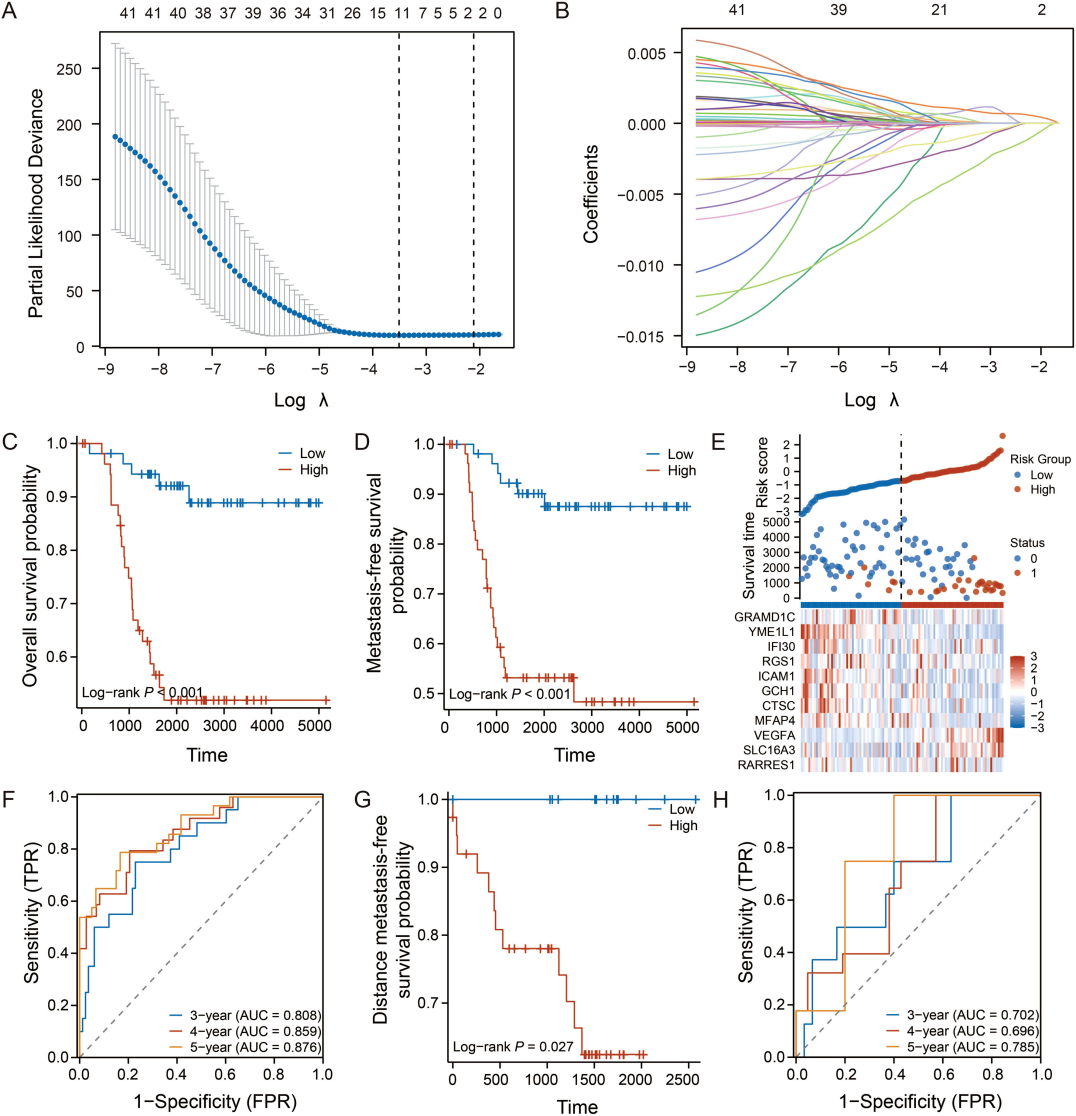
Construction and validation of the 11-gene prognostic signature. **(A, B)** LASSO Cox regression analysis identifying the optimal gene signature and their coefficients in the GSE58812 training cohort. **(C, D)** Kaplan-Meier curves for OS and MFS in the training cohort. **(E)** Heatmap showing the expression of the 11 signature genes, with corresponding risk score distribution and survival status for each patient. **(F)** Time-dependent ROC curves for 3-, 4-, and 5-year OS prediction in the training cohort. **(G, H)** Kaplan-Meier curve for distant metastasis-free survival (DMFS) and time-dependent ROC curves for the validation cohort (GSE53752). Log-rank test was used for survival analysis.

Based on the 42 TME-associated candidate genes, we employed LASSO-Cox regression analysis in the GSE58812 training cohort to construct a prognostic signature. An optimal 11-gene signature was established (**Figures 2A, B**). Using a formula derived from the regression coefficients (**Supplementary Table S4**), a risk score was calculated for each patient. Patients were then stratified into high- and low-risk groups based on the median score.

Kaplan-Meier analysis revealed that patients in the low-risk group exhibited significantly longer overall survival (OS) and metastasis-free survival (MFS) (*p* < 0.001, **Figures 2C, D**). The distribution of risk scores, survival status, and the 11-gene expression heatmap illustrated the clear stratification power of the model (**Figure 2E**). The time-dependent ROC analysis confirmed the excellent predictive performance of the signature, with the area under curve values for 3-, 4-, and 5-year OS exceeding 0.87 (**Figure 2F**).

To assess its robustness, the signature was validated in an independent cohort (GSE53752). Consistent with the training set, the model successfully stratified patients with distinct survival outcomes (*p* = 0.027, **Figure 2G**), and maintained favorable predictive accuracy (**Figure 2H**).

### 3.3 The prognostic signature reflects the tumor immune microenvironment landscape

**Figure 3:**
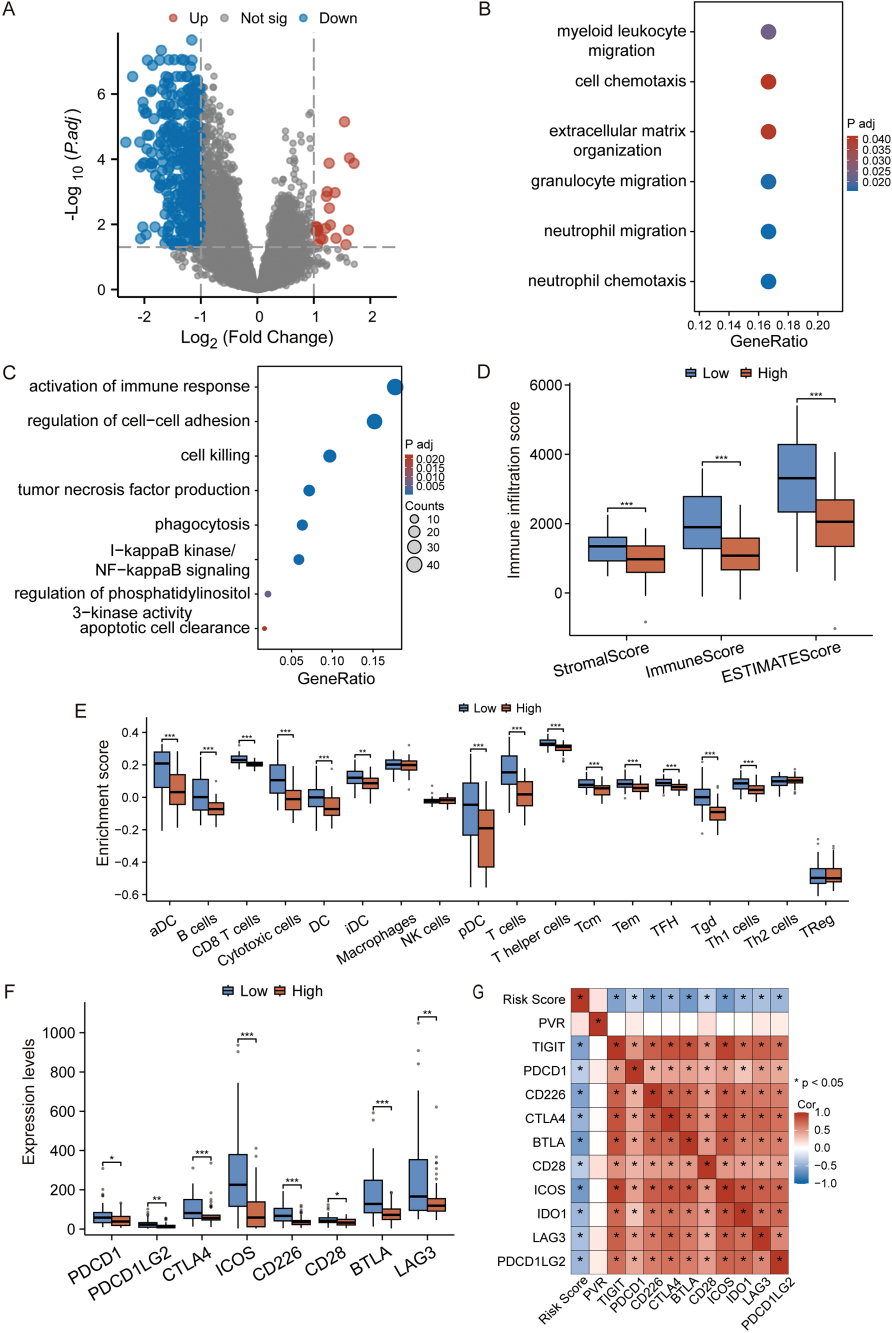
The prognostic signature reflects the tumor immune microenvironment. **(A)** Volcano plot of DEGs between high- and low-risk groups. Red and blue dots represent up- and down-regulated genes, respectively. Genes with an adjusted *p*-value ≤ 0.05 and |log2(Fold Change)| >1 are considered significant. **(B, C)** GO and KEGG enrichment analysis of genes upregulated in the high-risk **(B)** and low-risk **(C)** groups. **(D)** Comparison of Stromal, Immune, and ESTIMATE scores between risk groups. **(E)** ssGSEA analysis showing the relative enrichment of 28 immune cell subtypes. **(F)** Expression levels of key immune checkpoint genes in high- and low-risk groups. **(G)** The heatmap shows the correlation between the prognostic risk score and the expression level of immune checkpoint genes. Data in **(D-F)** are presented as box plots. *p < 0.05, **p < 0.01, ***p < 0.001 (Student’s t-test).

Given the TME-centric nature of our signature genes, we next investigated whether the risk score correlates with the TME characteristics in TNBC. We first identified DEGs between the high- and low-risk groups (**Figure 3A**). GO and KEGG analyses revealed that genes upregulated in the low-risk group were enriched in immune activation-related pathways, such as “regulation of cell-cell adhesion” and “cell killing,” whereas genes upregulated in the high-risk group were associated with “myeloid leukocyte migration” and “cell chemotaxis,” suggesting distinct immune states (**Figures 3B, C**).

To further substantiate this, we performed immune infiltration analysis. The ESTIMATE algorithm showed that the low-risk group had significantly higher Stromal, Immune, and overall ESTIMATE scores, indicating a more infiltrated, or “hot,” tumor microenvironment (**Figure 3D**). Furthermore, ssGSEA analysis revealed that the low-risk group was characterized by a higher infiltration of anti-tumor immune cells, including activated dendritic cells (aDC), CD8+ T cells, and T helper cells (**Figure 3E**).

Intriguingly, the expression of multiple immune checkpoint molecules, such as PDCD1 (PD-1), CTLA4, and LAG3, was also significantly elevated in the low-risk group (**Figure 3F**), suggesting a potentially “exhausted” yet targetable immune state. To further investigate whether the risk score has the predictive capacity for response to immune checkpoint inhibitors (ICIs) therapy, we evaluated its correlation with the expression of immune checkpoint molecules. It is worth noting that there is a significant negative correlations between the risk score and the mRNA levels of major inhibitory checkpoints (**Figure 3G**). This consistent inverse relationship indicates that patients in the low-risk group, despite having a more robust immune infiltration, also exhibit a higher degree of immune checkpoint expression, a hallmark of T-cell exhaustion. This specific immune contexture—characterized by the coexistence of cytotoxic immune cells and elevated checkpoint levels—is often associated with a heightened likelihood of responding to immune checkpoint blockade therapy.

### 3.4 MFAP4 is pinpointed as a key tumor suppressor within the signature

**Figure 4:**
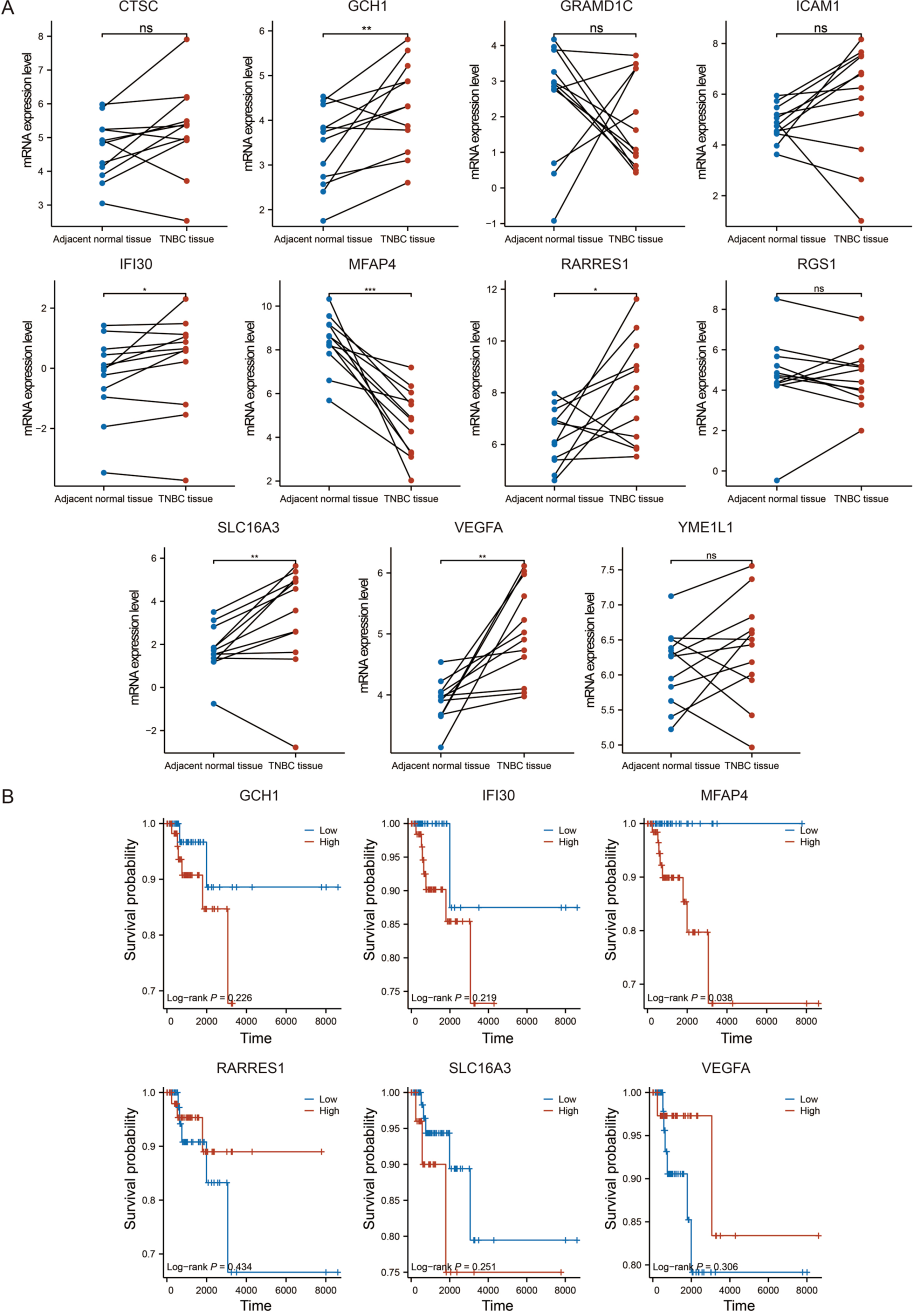
Identification of MFAP4 as the key prognostic gene. **(A)** Expression levels of the 11 signature genes in paired TNBC and adjacent normal tissues from the TCGA-BRAC dataset. **(B)** Kaplan-Meier survival analysis for six selected genes in the TCGA-TNBC cohort. Paired Student’s t-test **(A)** and log-rank test **(B)** were used. ns, not significant. *p < 0.05, **p < 0.01, ***p < 0.001; ns, not significant.

To pinpoint the most promising therapeutic target among the 11 signature genes, we implemented a multi-step screening strategy. First, analysis of paired TNBC and adjacent normal tissues from the TCGA-BRAC dataset revealed that six genes were significantly dysregulated (**Figure 4A**). Among them, we focused on those whose low expression might predict worse outcomes, consistent with a tumor-suppressive role. Subsequent Kaplan-Meier analysis of these candidates in the TCGA-TNBC cohort demonstrated that only low expression of MFAP4 was significantly associated with poorer OS (*p* = 0.038, **Figure 4B**). Crucially, as an ECM protein, MFAP4’s biological identity perfectly aligns with the TME-centric nature of our prognostic model. This, combined with its strong prognostic value and the fact that its specific function in TNBC remains uncharacterized, established MFAP4 as the top candidate for in-depth functional investigation.

### 3.5 Overexpression of MFAP4 suppresses TNBC cell proliferation and migration *in vitro*

**Figure 5:**
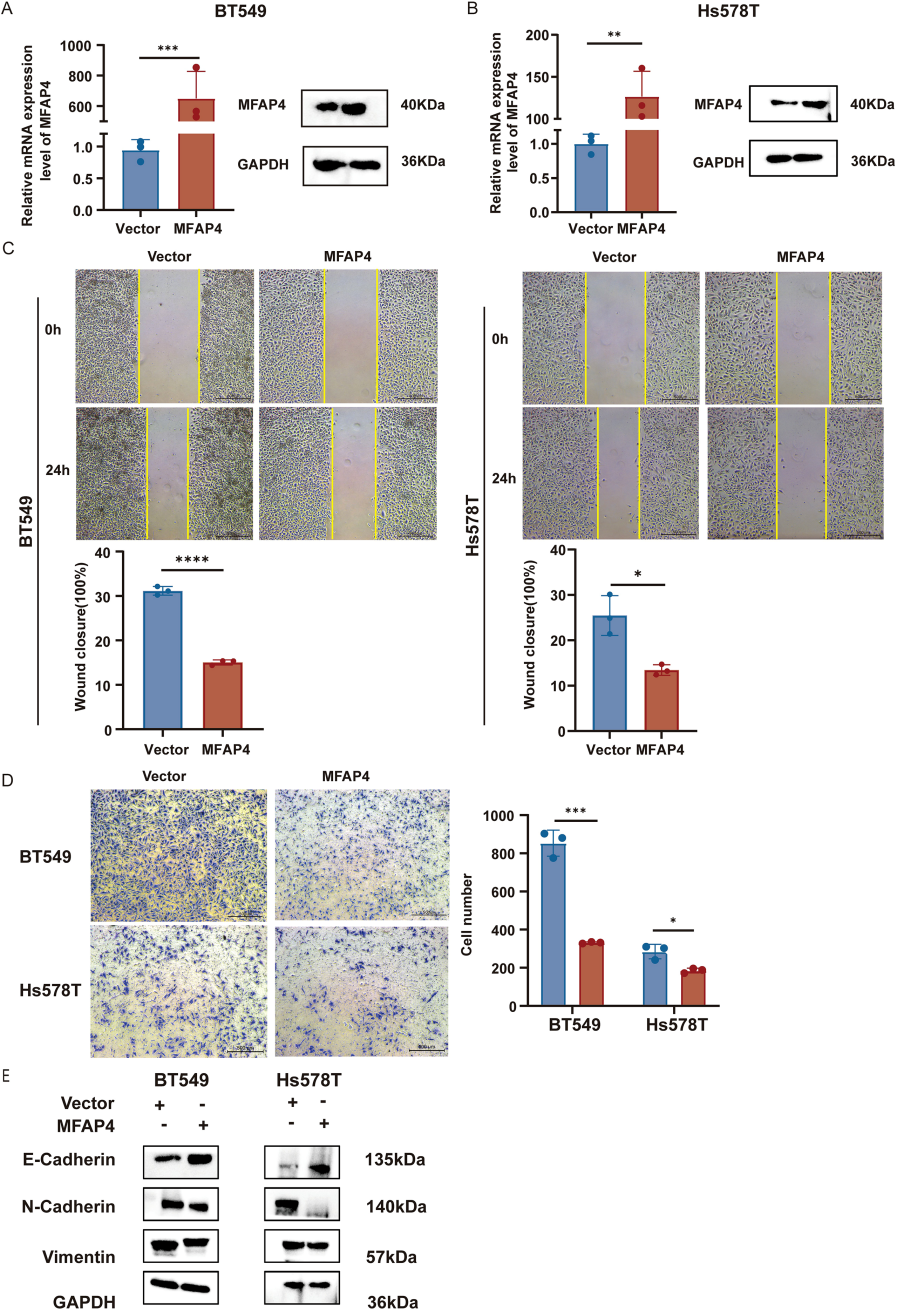
MFAP4 overexpression inhibits TNBC cell migration. **(A, B)** Validation of MFAP4 overexpression in BT-549 and Hs-578T cells by RT-qPCR and WB. **(C)** Wound healing assays showing impaired migration in MFAP4-overexpressing cells at 24 (h) Scale bar, 500 μm. **(D)** Transwell migration assays showing reduced migratory cell numbers after 16 (h) Scale bar, 500 μm. **(E)** WB demonstrates expression levels of key EMT markers in MFAP4-overexpressing versus control groups. Data are expressed as mean ± SD. Each data point in the statistical graph represents an independent repeated experiment, with n=3 independent experiments.*p < 0.05, **p < 0.01, ***p < 0.001, ****p < 0.0001 (Student’s t-test).

To investigate the biological function of MFAP4 in TNBC, we established stable MFAP4-overexpressing cell lines in two TNBC cell lines, BT-549 and Hs-578T. The overexpression efficiency was robustly confirmed at both mRNA and protein levels (**Figures 5A, B**).

We first assessed the impact of MFAP4 on cell migration. Wound healing assays demonstrated that MFAP4 overexpression significantly impaired the migratory capacity of both BT-549 and Hs-578T cells (**Figure 5C**). This finding was corroborated by Transwell migration assays, which showed a marked reduction in the number of migrating cells in the MFAP4-overexpressing groups (**Figure 5D**). To further explore the mechanism behind the impaired migration, we examined key markers of epithelial-mesenchymal transition (EMT). WB analysis revealed that MFAP4 overexpression led to a significant upregulation of the epithelial marker E-cadherin, and a concomitant downregulation of the mesenchymal markers N-cadherin and Vimentin (**Figure 5E**). This indicates that MFAP4 may suppress TNBC cell migration, at least in part, by inhibiting the EMT process.

**Figure 6:**
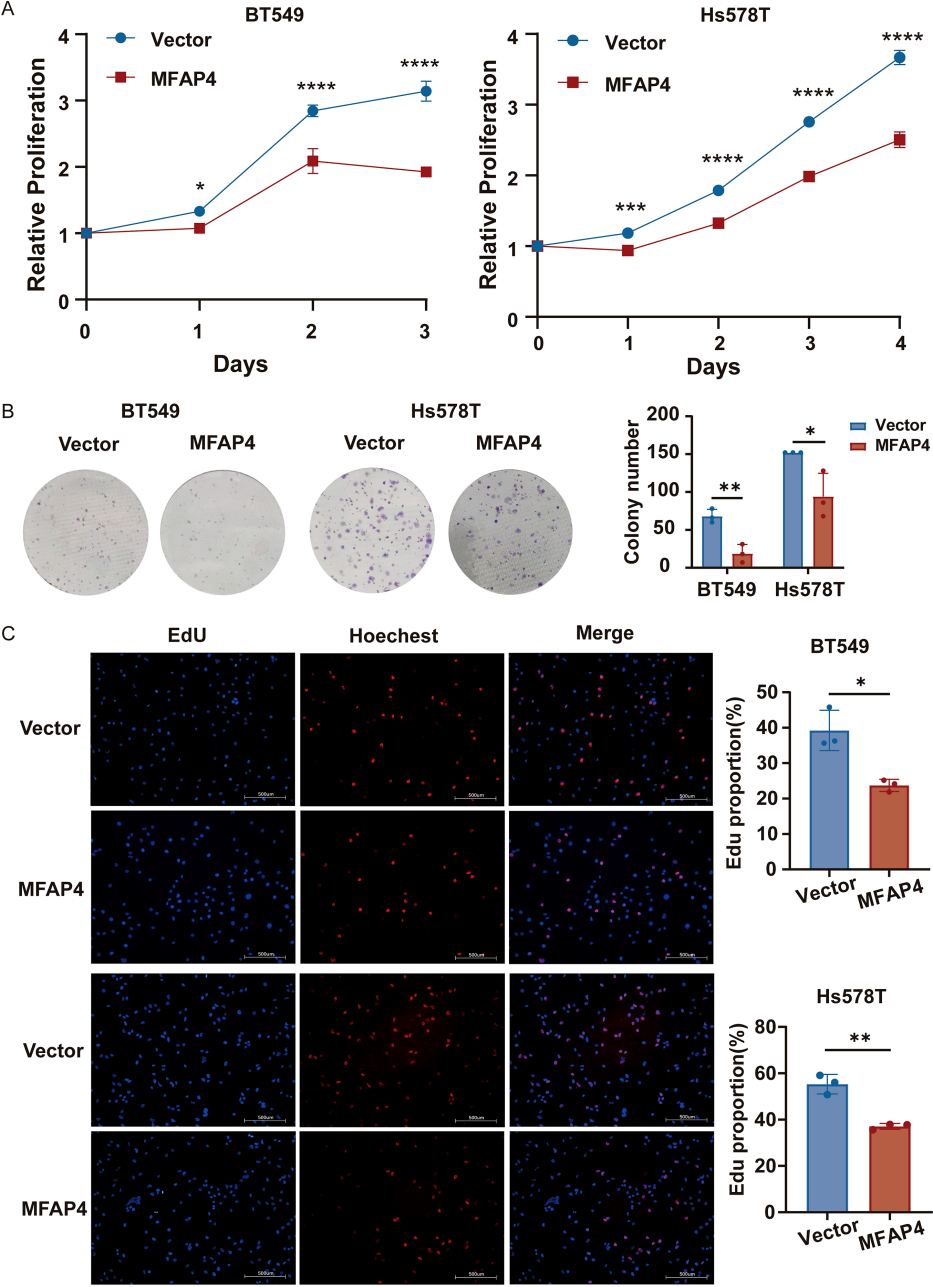
MFAP4 overexpression suppresses TNBC cell proliferation. **(A)** CCK-8 proliferation curves for vector and MFAP4-overexpressing cells. **(B)** Colony formation assays showing reduced colony numbers after 10–14 days. **(C)** EdU incorporation assays showing a decreased percentage of proliferating cells. Scale bar, 500 μm. Data are expressed as mean ± SD. Each data point in the statistical graph represents an independent repeated experiment, with n=3 independent experiments. *p < 0.05, **p < 0.01, ***p < 0.001, ****p < 0.0001 (Student’s t-test).

Next, we evaluated the effect of MFAP4 on cell proliferation. CCK-8 assays revealed that MFAP4 overexpression significantly inhibited cell growth over a 4-day period (**Figure 6A**). Consistent with this, colony formation assays showed that MFAP4-overexpressing cells formed significantly fewer and smaller colonies (**Figure 6B**). Furthermore, EdU incorporation assays confirmed that MFAP4 overexpression led to a significant decrease in the proportion of cells undergoing DNA synthesis (**Figure 6C**). Collectively, these results provide strong evidence that MFAP4 functions as a tumor suppressor in TNBC by inhibiting key malignant phenotypes.

### 3.6 MFAP4 exerts its tumor-suppressive function by inhibiting the PI3K/AKT/mTOR pathway

**Figure 7:**
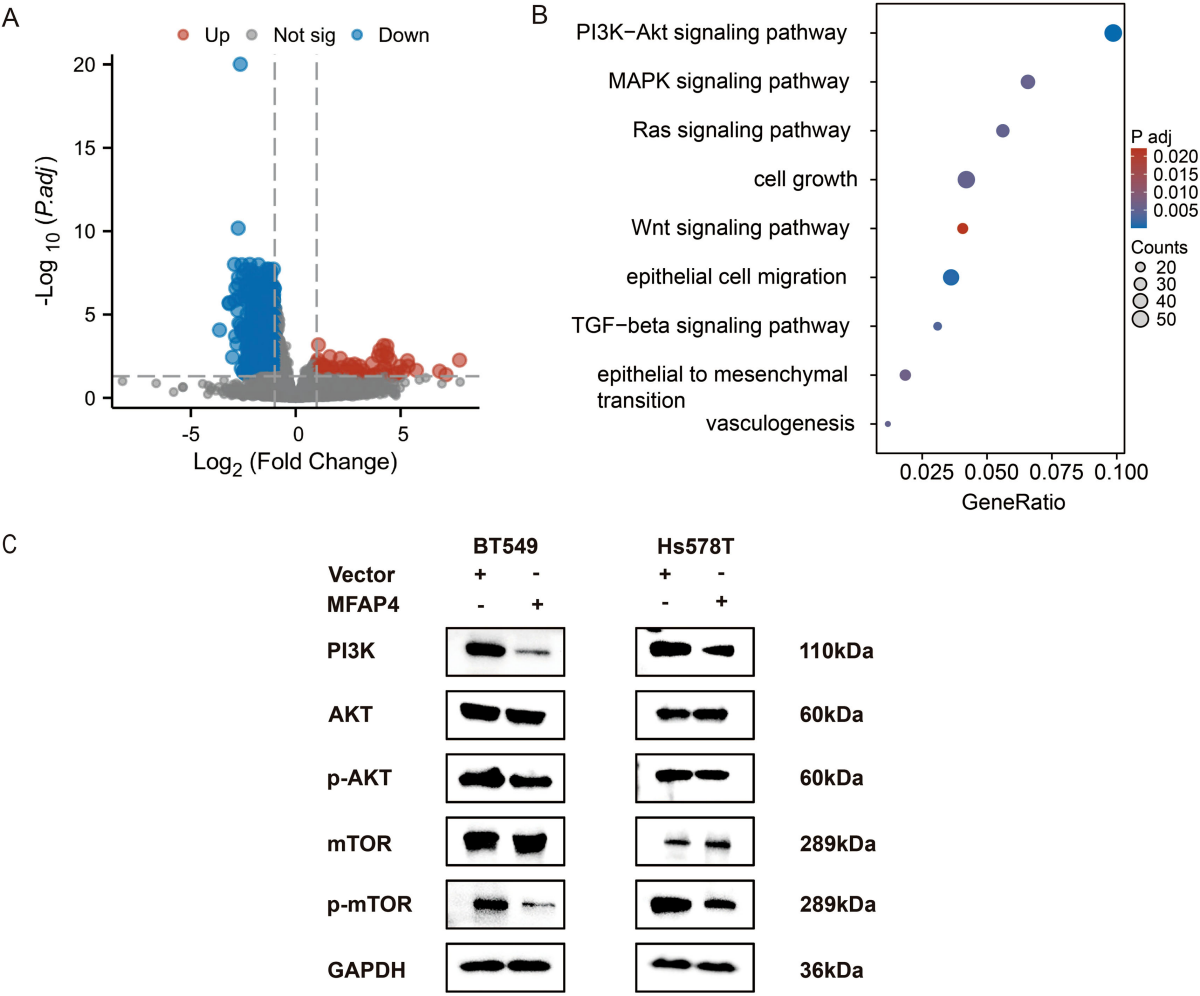
MFAP4 negatively regulates the PI3K/AKT/mTOR pathway. **(A)** Volcano plot of DEGs between MFAP4-high and MFAP4-low expression groups in the TCGA-TNBC cohort. Red and blue dots represent up- and down-regulated genes, respectively. Genes with an adjusted *p*-value ≤ 0.05 and |log2(Fold Change)| > 1 are considered significant. **(B)** KEGG pathway analysis of the DEGs, highlighting the enrichment of the PI3K-AKT signaling pathway in the MFAP4-low group. **(C)** WB analysis showing the phosphorylation levels of key pathway components (PI3K, AKT, p-AKT, mTOR, p-mTOR) in MFAP4-overexpressing vs. vector control BT-549 and Hs-578T cells. GAPDH served as a loading control.

To elucidate the molecular mechanism underlying MFAP4’s tumor-suppressive effects, we leveraged transcriptome data from the TCGA-TNBC cohort. Patients were stratified into “MFAP4-high” and “MFAP4-low” expression groups based on the median expression level of MFAP4. Differential expression analysis between these two groups identified 70 upregulated and 991 downregulated genes in the MFAP4-high group (**Figure 7A**). To identify the signaling pathways potentially regulated by MFAP4 in a clinical context, we performed KEGG pathway analysis on these DEGs. Remarkably, the results showed that genes positively correlated with MFAP4 expression (i.e., downregulated in the MFAP4-low group) were significantly enriched in pathways that suppress tumorigenesis, while genes negatively correlated with MFAP4 were enriched in key oncogenic signaling cascades. The PI3K-AKT signaling pathway emerged as one of the most significantly enriched pathways associated with low MFAP4 expression (**Figure 7B**).

This bioinformatic finding from patient tissues, combined with the well-established role of PI3K/AKT/mTOR in promoting TNBC progression (16), led us to hypothesize that MFAP4 might exert its function by inhibiting this axis. To directly test this causal relationship at the cellular level, we performed WB analysis on our engineered cell lines. As hypothesized, stable overexpression of MFAP4 in BT-549 and Hs-578T cells significantly reduced the expression levels of key pathway activation markers, including PI3K (p110α) as well as phosphorylated AKT (Ser473) and mTOR (Ser2448), without affecting total AKT or mTOR protein levels (**Figure 7C**).

**Figure 8:**
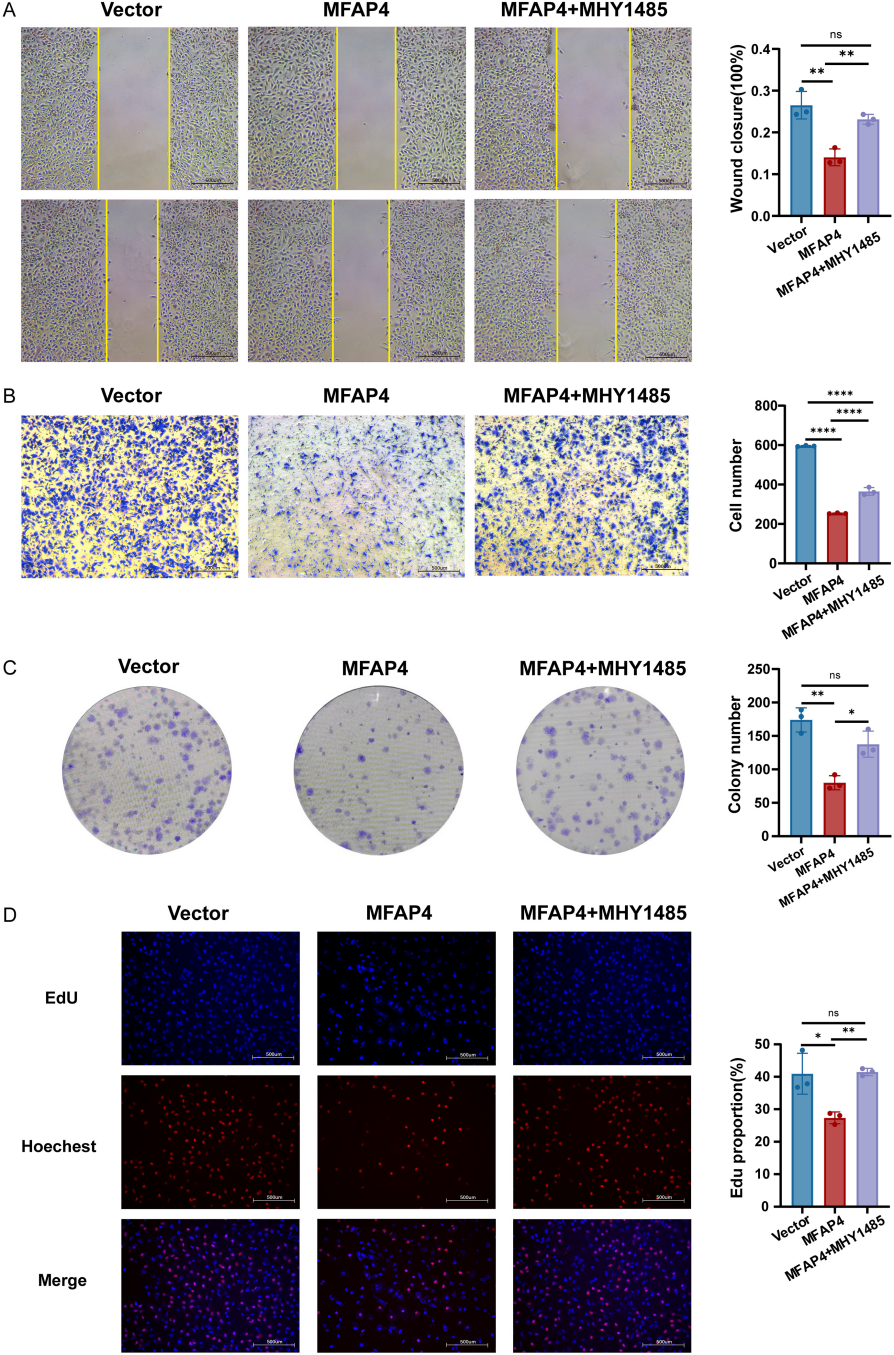
Pharmacological activation of mTOR rescues the anti-tumor effects of MFAP4. Hs-578T cells (Vector or MFAP4-overexpressing) were treated with vehicle or mTOR agonist MHY1485 (3 μM) for 24 (h) **(A)** Wound healing and **(B)** Transwell assays showing reversal of migration inhibition. Scale bar, 500 μm. **(C)** Colony formation and **(D)** EdU assays showing reversal of proliferation suppression. Scale bar, 500 μm. Data are expressed as mean ± SD. Each data point in the statistical graph represents an independent repeated experiment, with n=3 independent experiments. *p < 0.05, **p < 0.01, ****p < 0.0001, ns, not significant (One-way ANOVA with Tukey’s *post hoc* test).

**Figure 9:**
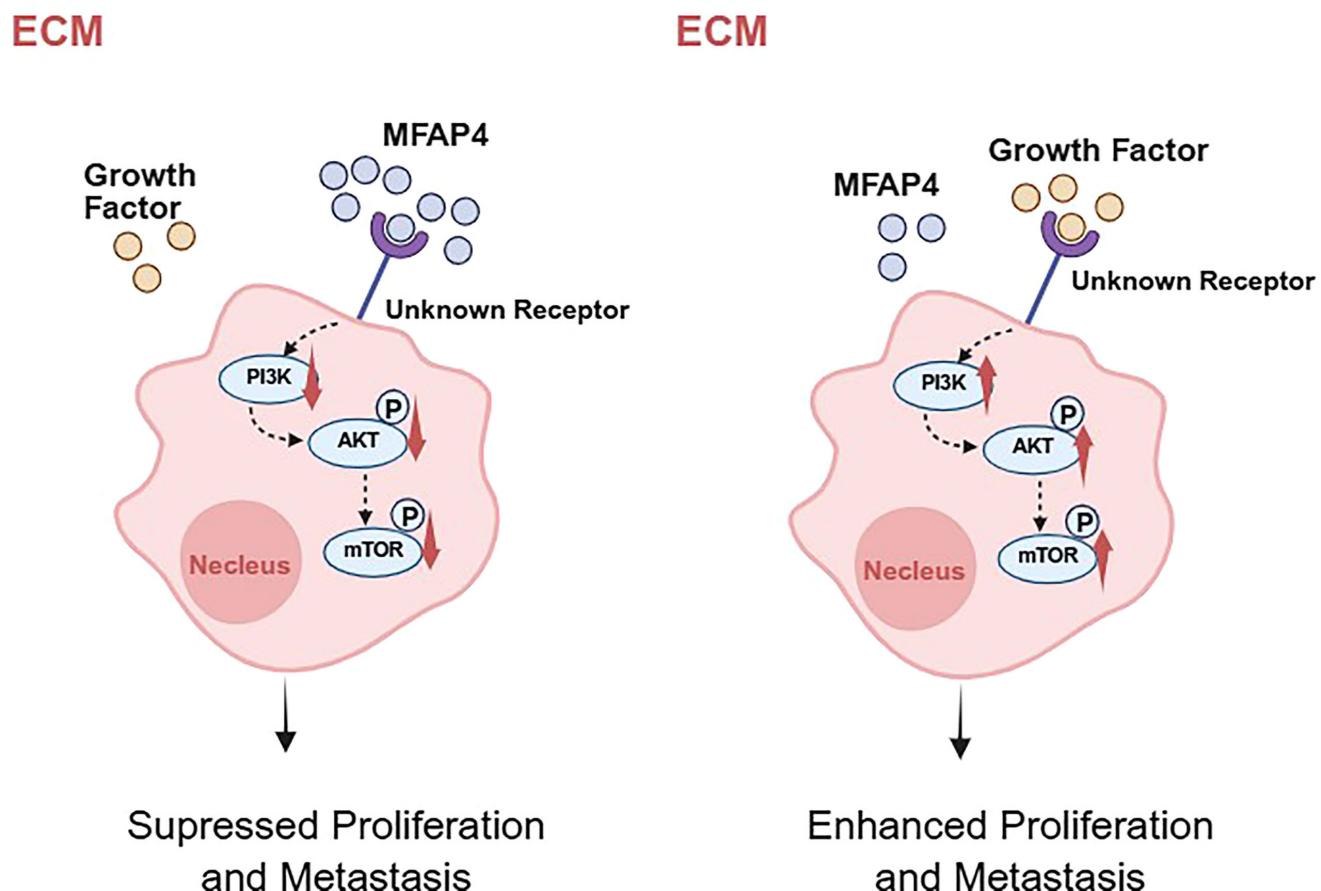
A hypothetical working model for MFAP4-mediated suppression of TNBC progression. Based on our data and existing literature, we propose a hypothetical model. In TNBC cells with high MFAP4 expression (left), secreted MFAP4 may bind to an unknown cell surface receptor, potentially modulating its signaling activity. This leads to the suppression of the intracellular PI3K/AKT/mTOR signaling cascade, thereby inhibiting cell proliferation and migration. Conversely, in TNBC cells with low MFAP4 expression (right), the proposed receptor is not bound by MFAP4 and may be activated by other ligands, leading to hyperactivation of the PI3K/AKT/mTOR pathway and promotion of a malignant phenotype. The precise identity of the receptor in TNBC remains to be experimentally confirmed. Created with BioRender.com.

To functionally validate this mechanism, we conducted a rescue experiment using MHY1485, a potent mTOR agonist. Crucially, treatment with MHY1485 significantly reversed the inhibitory effects of MFAP4 overexpression on cell migration, as demonstrated by both wound healing and Transwell assays (**Figures 8A, B**). Similarly, MHY1485 treatment also partially rescued the proliferation defect caused by MFAP4, restoring colony formation ability and increasing the proportion of EdU-positive cells (**Figures 8C, D**). These findings, integrating clinical cohort analysis with direct experimental validation, mechanistically link MFAP4 to the PI3K/AKT/mTOR pathway, demonstrating that MFAP4 suppresses TNBC malignancy, at least in part, by restraining this critical oncogenic cascade (**Figure 9**).

## 4 Discussion

TNBC remains a significant clinical challenge due to its aggressive nature and limited therapeutic avenues (17). In this study, we address this challenge by not only developing a robust, TME-informed prognostic signature but also by uncovering a novel regulatory axis that links the extracellular matrix directly to intracellular oncogenic signaling. We identify the previously underexplored ECM protein, MFAP4, as a key tumor suppressor in TNBC that functions by restraining the PI3K/AKT/mTOR pathway, a cornerstone of TNBC malignancy. This discovery provides a new layer of understanding of TME-tumor cell crosstalk and presents MFAP4 as a promising biomarker and therapeutic target.A key strength of our prognostic signature lies in its ability to reflect the underlying immune landscape of TNBC. We observed that the low-risk group, associated with better survival, was characterized by a “hot” or immune-infiltrated microenvironment, with elevated levels of cytotoxic lymphocytes. Intriguingly, this immune-active state was accompanied by higher expression of immune checkpoint molecules like PD-1 and CTLA4. Meanwhile, relevant analyses show that the risk score itself is negatively correlated with the expression of these checkpoint molecules. This seemingly paradoxical finding is consistent with the concept of an “active but exhausted” immune response, a state often predictive of a favorable response to ICIs. Therefore, our 11-gene signature holds potential for significant clinical translation. Beyond just predicting prognosis, it could serve as a non-invasive biomarker to identify a subset of TNBC patients who might derive the most benefit from immunotherapy, thus guiding personalized treatment decisions. This addresses a critical need in TNBC therapy, where reliable biomarkers for ICIs efficacy are still scarce.

Our study brings MFAP4, a molecule with a history of context-dependent roles in cancer, into the spotlight of TNBC research. While previously reported as pro-tumorigenic in some sarcomas, our findings unequivocally position MFAP4 as a tumor suppressor in TNBC. This context-dependency is a common feature of ECM proteins, whose functions can be dictated by the specific cellular milieu, interacting partners, or post-translational modifications unique to each cancer type (18). A key aspect of TNBC aggressiveness is its propensity for invasion and metastasis, a process heavily reliant on EMT. Our results provide a novel mechanistic layer to MFAP4’s anti-migratory function, demonstrating that its overexpression reverses key EMT hallmarks. This suggests that the loss of MFAP4 in the tumor microenvironment may be a permissive event that facilitates the EMT program, thereby empowering TNBC cells to migrate and invade. Furthermore, our results align with previous reports suggesting MFAP4’s involvement in immune regulation, such as inducing immune cell chemotaxis (19, 20). This raises a fascinating possibility that MFAP4 might suppress TNBC progression through a dual mechanism. On one hand, it directly inhibits tumor cell intrinsic malignant properties by restraining EMT and the PI3K/AKT pathway. On the other hand, it may actively remodel the TME into a more anti-tumor state by recruiting beneficial immune cells, a hypothesis that aligns perfectly with the immune-hot phenotype of the MFAP4-high/low-risk group identified by our model.

The mechanistic core of our study is the discovery of the MFAP4-PI3K/AKT/mTOR axis. The PI3K/AKT pathway is notoriously hyperactivated in a majority of TNBC cases, making it a prime therapeutic target (16, 21). The development of inhibitors like ipatasertib has shown promise, but identifying which patients will respond remains a challenge (22, 23). Our findings provide a crucial piece of this puzzle. We propose that the loss of MFAP4 expression in the TME could be a key event that unleashes the PI3K/AKT pathway, driving TNBC aggressiveness. This establishes MFAP4 not just as a downstream consequence of cancer, but as an upstream regulator.

A critical question remains: how does an extracellular protein like MFAP4 communicate with the intracellular PI3K/AKT pathway? While the definitive identification of the specific receptor in TNBC is beyond the scope of this study, our proposed model is informed by existing literature. Previous studies have established that MFAP4 can act as a ligand for specific integrins in other biological contexts (24). Given that integrins are effective activators of the PI3K/AKT pathway (25), we hypothesize that in TNBC, MFAP4 may exert its tumor-suppressive effect by engaging similar integrin receptors. This interaction can transduce signals that inhibit the PI3K/AKT/mTOR cascade or compete with other tumor-promoting ligands for receptor binding. Alternatively, MFAP4 may competes for binding with these growth factors in the ECM, thereby preventing their interaction with receptor tyrosine kinases (RTKs) upstream of PI3K (26). This model, which bridges the extracellular space with intracellular oncogenic signaling, opens new avenues for therapeutic intervention.

The clinical implications of our findings are profound. First, MFAP4 itself could be a valuable biomarker. Low MFAP4 levels in tumor tissue or even in circulation (as MFAP4 is a secreted protein) could identify high-risk TNBC patients who may require more aggressive treatment or be candidates for PI3K/AKT inhibitors. Second, and more ambitiously, restoring MFAP4 function represents a novel therapeutic strategy. This could be achieved through recombinant MFAP4 protein therapy or gene therapy approaches. Such a strategy would be fundamentally different from current inhibitors, as it aims to restore a natural braking mechanism rather than just blocking an overactive engine, potentially leading to fewer off-target effects.

While our study provides a solid foundation, it has several limitations that pave the way for future research. First and foremost, the prognostic model, while rigorously validated using multiple public datasets, has not yet been tested in an independent, real-world clinical cohort. This is a significant limitation that currently restricts its direct clinical applicability. The gold standard for clinical translation requires validating the signature’s performance using assays such as RT-qPCR or immunohistochemistry on a well-characterized set of patient samples, ideally from a prospective study. Therefore, securing this validation constitutes a critical prerequisite for clinical adoption and is a central objective of our subsequent research.

Another notable limitation of this study is that the functional insights for MFAP4 are derived solely from *in vitro* cell-based assays. While we obtained consistent and compelling data across two distinct TNBC cell lines, the absence of *in vivo* validation in animal models limits the generalizability of our findings. The *in vivo* milieu, which encompasses complex tumor-microenvironment interactions, cannot be fully recapitulated *in vitro.* Therefore, employing *in vivo* models to confirm the tumor-suppressive role of MFAP4 at the organismal level will be a crucial next step to solidify our conclusions and advance their translational potential.

Furthermore, although we enhanced the functional rescue experiment using the mTOR agonist MHY1485 and demonstrated that reactivating the PI3K/AKT/mTOR pathway could counteract the tumor suppressive effect of MFAP4, we acknowledged the possibility of off-target effects, which is a common limitation of kinase-targeted compounds. Therefore, although our data strongly suggest the PI3K/AKT/mTOR axis, to clearly confirm this connection, ideally, more specific genetic methods are needed, such as expressing AKT or mTOR that constitutes the active form, or using siRNA to verify pathway dependence. These experiments represent an important direction for future research, aiming to dissect this new signal axis more precisely.

Meanwhile, in the future, we will further refine the working model we hypothesized - MFAP4 signals through specific cell surface receptors - which requires direct experimental verification. The application of unbiased screening techniques, such as co-immunoprecipitation mass spectrometry using recombinant MFAP4, is crucial for the clear identification of cell surface receptors mediating MFAP4 anti-tumor signaling in TNBC cells.

In conclusion, this study presents a substantial advance by identifying a novel link between TME and a critical intracellular oncogenic pathway. We have identified MFAP4, a previously underexplored extracellular matrix protein, as a key tumor suppressor in TNBC. Our mechanistic investigation further reveals that MFAP4 exerts this function by restraining the PI3K/AKT/mTOR pathway, a pivotal driver of TNBC malignancy. This connection between an ECM component and intracellular oncogenic signaling is significant, offering new insights into tumor progression and potential therapeutic vulnerabilities. In parallel, our work establishes a robust 11-gene prognostic signature, validated across multiple large cohorts, which effectively stratifies TNBC patients and is deeply rooted in the biology of the TME.

## Data Availability

The original contributions presented in the study are included in the article/**Supplementary Materials**, further inquiries can be directed to the corresponding author/s.
