# Supplementary material for: A novel prognostic signature identifies MFAP4 as a tumor suppressor linking the tumor microenvironment to PI3K/AKT signaling in triple-negative breast cancer
Source: Front Immunol. 2025 Dec 10;16:1709141. doi: 10.3389/fimmu.2025.1709141 (PMC12727647; doi:10.3389/fimmu.2025.1709141)
Supplement: Supplementary file 1 [file Table1.docx]

**Supplementary Table S1:** **Summary of the public databases used in this study. The table includes the dataset ID, source, tumor type, platform, sample size, and its purpose in the study.**

| Dataset ID | Source | Tumor Type | Platform | Sample Size | Purpose |
| --- | --- | --- | --- | --- | --- |
| GSE5327 | GEO | Breast Cancer | GPL96 [HG-U133A] Affymetrix Human Genome U133A Array | 58 | Screening (WGCNA) |
| GSE65194 | GEO | Breast Cancer | GPL570 [HG-U133_Plus_2] Affymetrix Human Genome U133 Plus 2.0 Array | 178 | Screening (DEGs) |
| GSE58812 | GEO | TNBC | GPL570 [HG-U133_Plus_2] Affymetrix Human Genome U133 Plus 2.0 Array | 107 | Training Cohort |
| GSE53752 | GEO | TNBC | GPL7264 Agilent-012097 Human 1A Microarray (V2) G4110B (Probe Name version) | 51 | Validation Cohort |
| TCGA-BRCA | TCGA | TNBC | Illumina HiSeq RNA-Seq | 118 | Target Gene Screening |

**Supplementary Table S2: Sequences of oligonucleotide primers used for quantitative real-time PCR. For each gene, the table lists the gene symbol, gene name, forward primer sequence (5‘ to 3’), and reverse primer sequence (5‘ to 3’).**

| Gene Symbol | Gene Name |  | Sequence (5’→3’) |
| --- | --- | --- | --- |
| *MFAP4* (Human) | microfibril associated protein 4 | Forward | 5′‐TGACTACAAGCTGGGTTCG‐3′ |
|  |  | Reverse | 5ʹ‐CAGTGTCAGGAGGTGCATGT‐3′ |
| *GAPDH* (Human) | glyceraldehyde-3-phosphate dehydrogenase | Forward | 5′‐GGAGCGAGATCCCTCCAAAAT‐3′ |
|  |  | Reverse | 5ʹ‐GGCTGTTGTCATACTTCTCATGG‐3′ |

**Supplementary Table S3: Antibodies for Western blot. This table specifies the antibody name, host type, catalog number, supplier, and the dilution used for each application.**

| Antibody | Host | Catalog | Suppliers | Dilution Ratio |
| --- | --- | --- | --- | --- |
| MFAP4 | Mouse | A-9 | Santa Cruz | 1:500 |
| GAPDH | Mouse | 60004-1- Ig | Proteintech | 1:8000 |
| E-Cadherin | Mouse | 60335-1-Ig | Proteintech | 1:1000 |
| N-Cadherin | Rabbit | 13116 | Cell Signaling Technology | 1:1000 |
| Vimentin | Rabbit | 5741 | Cell Signaling Technology | 1:1000 |
| P110 PI3K | Rabbit | C73F8 | Cell Signaling Technology | 1:1000 |
| AKT(pan) | Rabbit | 4691S | Cell Signaling Technology | 1:1000 |
| Phospho-AKT (Ser473) | Rabbit | 4060S | Cell Signaling Technology | 1:1000 |
| mTOR | Mouse | 66888 | Proteintech | 1:1000 |
| Phospho-mTOR (Ser2448) | Mouse | 67778 | Proteintech | 1:1000 |
| HRP-Goat Anti-Rabbit Recombinant Secondary Antibody | Goat | RGAR001 | Proteintech | 1:5000 |
| HRP-Goat Anti-Mouse Recombinant Secondary Antibody | Goat | RGAM001 | Proteintech | 1:5000 |
