# Supplementary material for: A novel prognostic signature identifies MFAP4 as a tumor suppressor linking the tumor microenvironment to PI3K/AKT signaling in triple-negative breast cancer
Source: Front Immunol. 2025 Dec 10;16:1709141. doi: 10.3389/fimmu.2025.1709141 (PMC12727647; doi:10.3389/fimmu.2025.1709141)

Unedited blot images

Full unedited gel for Figure 5A

MFAP4

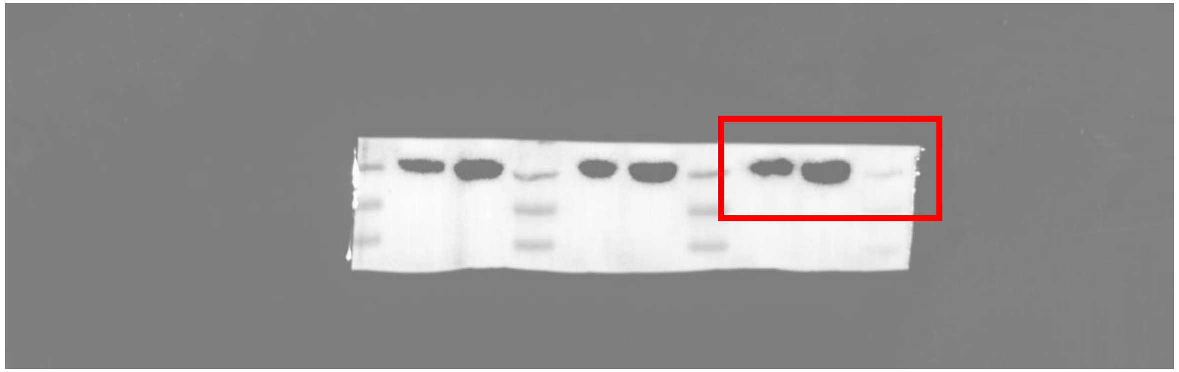

GAPDH

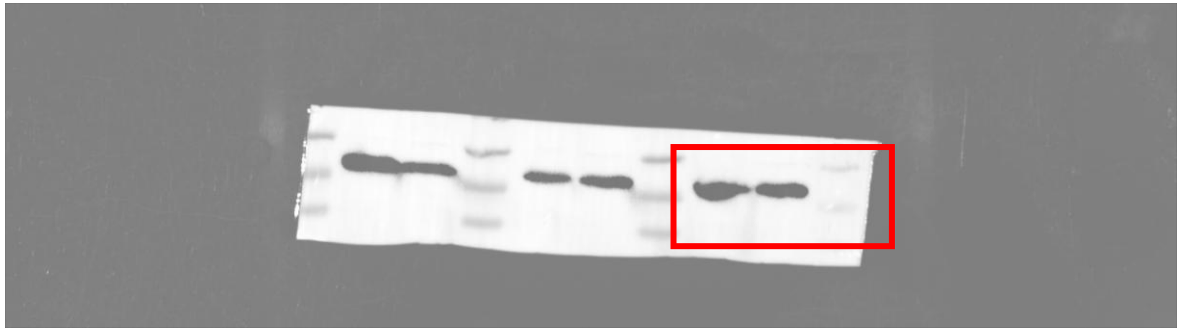

Full unedited gel for Figure 5B

MFAP4

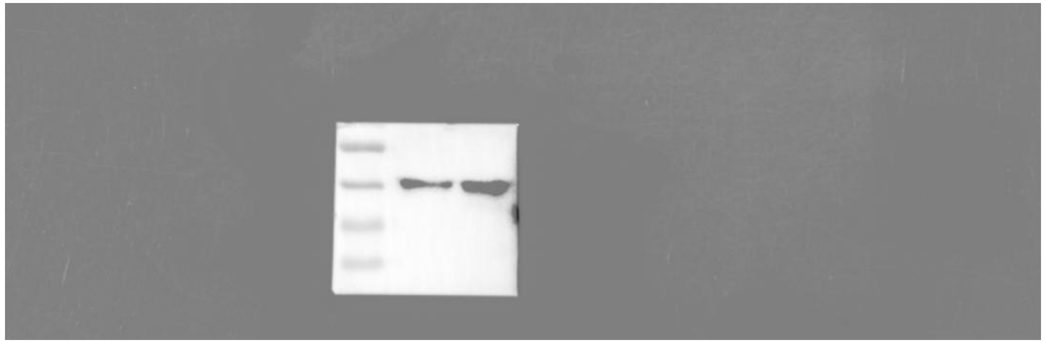

GAPDH

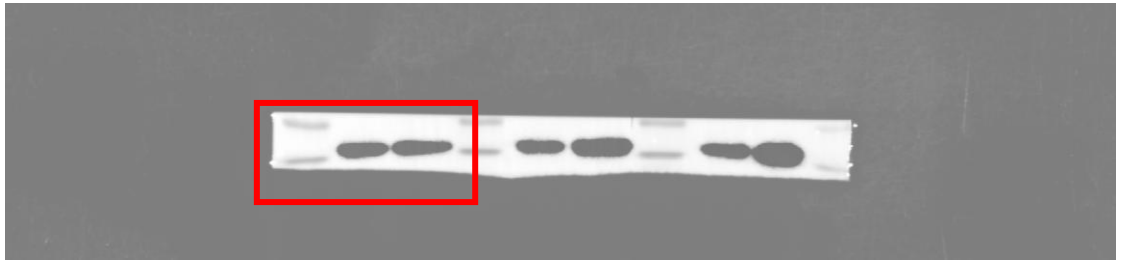

Full unedited gel for Figure 5E——BT549

E-cadherin

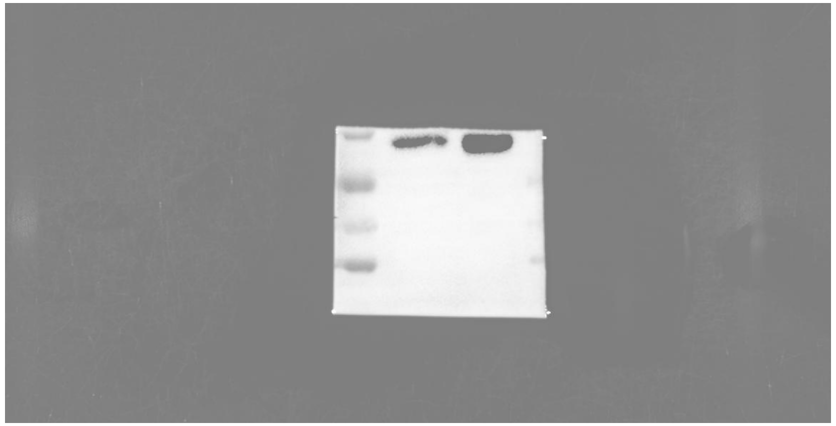

Vimentin

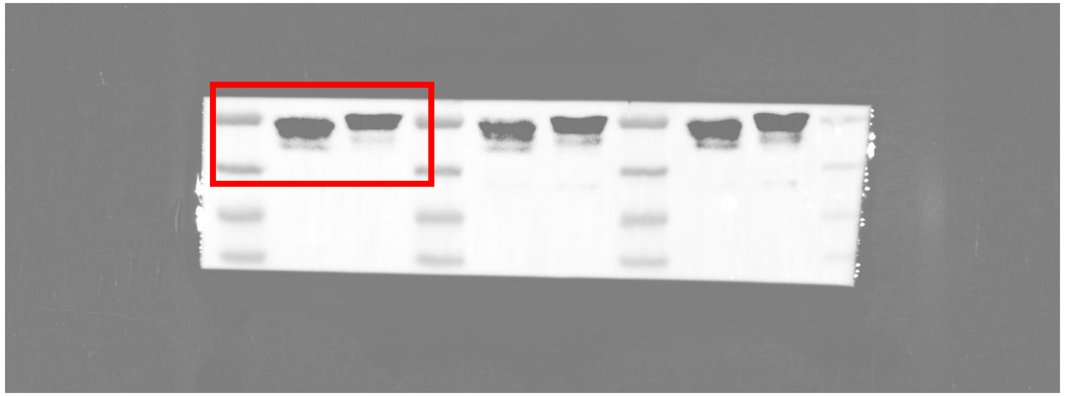

N-cadherin

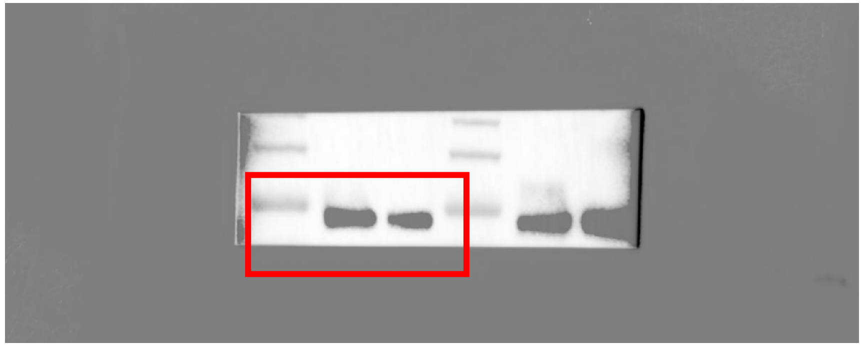

GAPDH

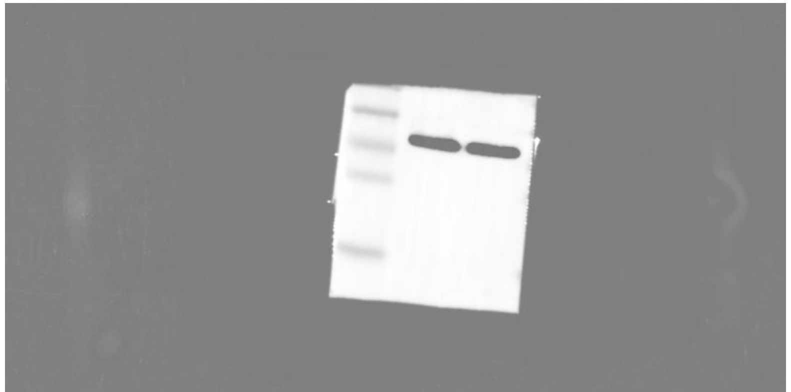

Full unedited gel for Figure 5E——Hs578T

E-cadherin

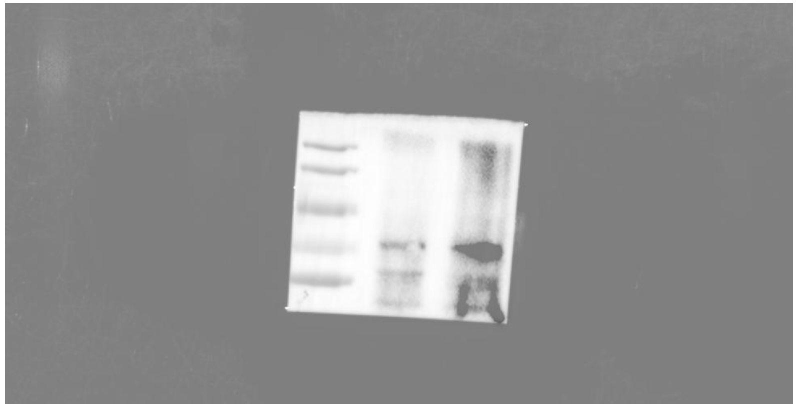

Vimentin

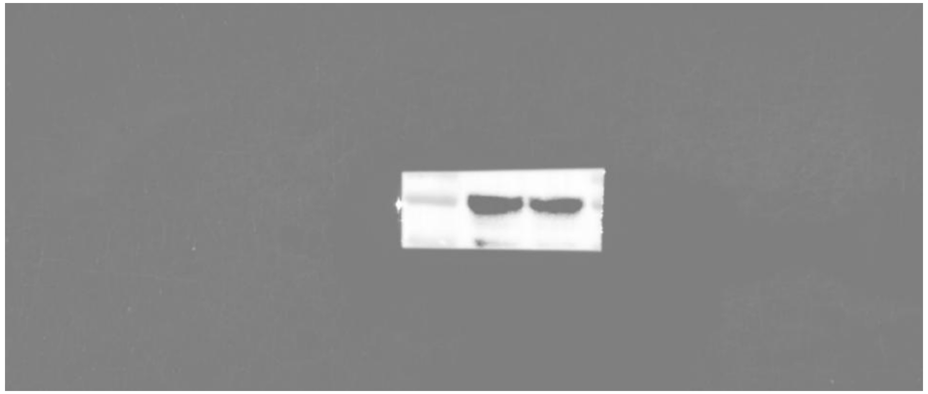

N-cadherin

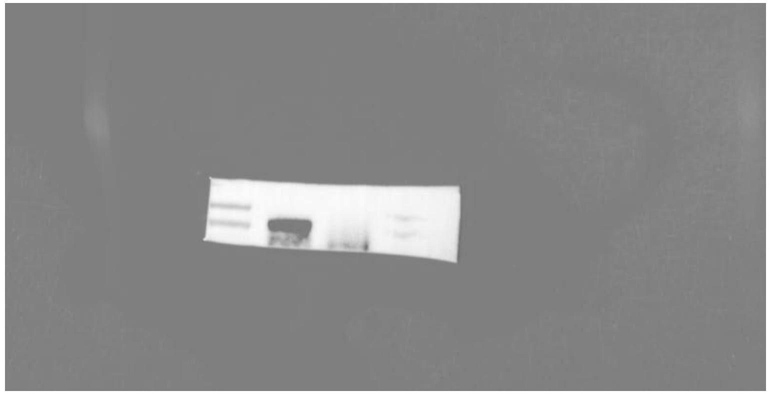

GAPDH

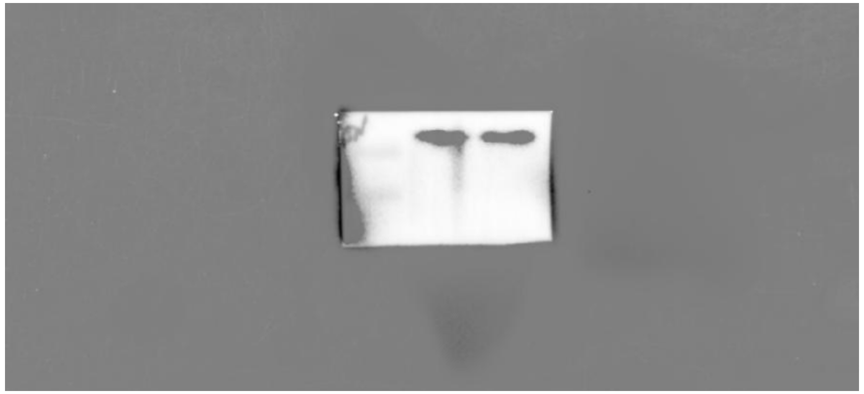

Full unedited gel for Figure 7C——BT549

PI3K

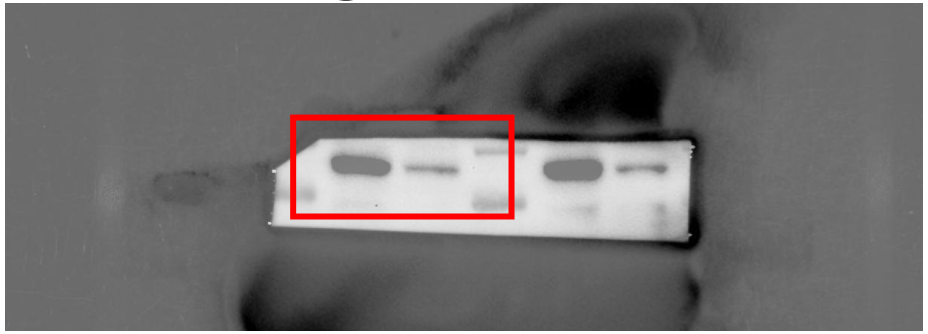

AKT

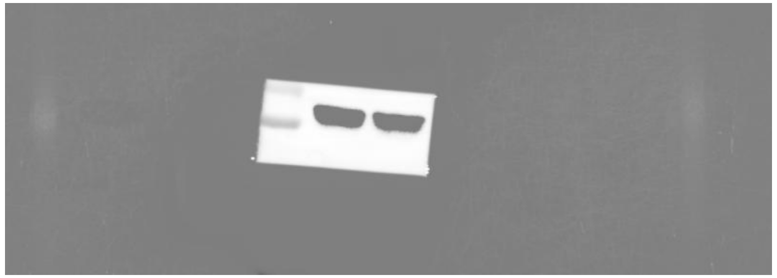

mTOR

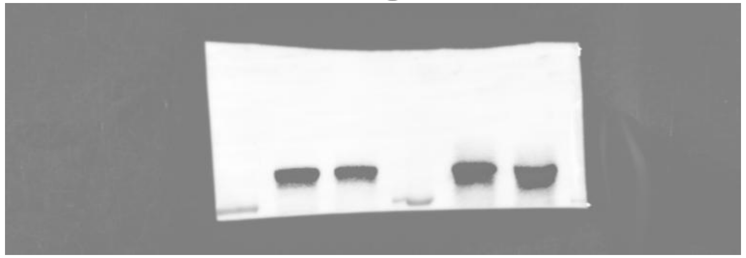

p-AKT

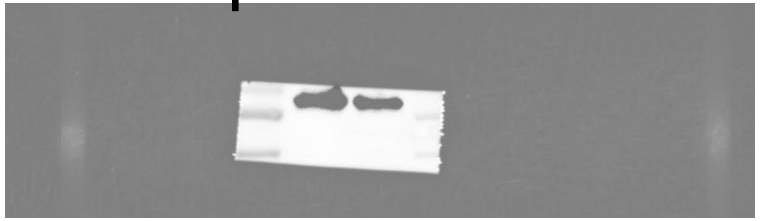

p-mTOR

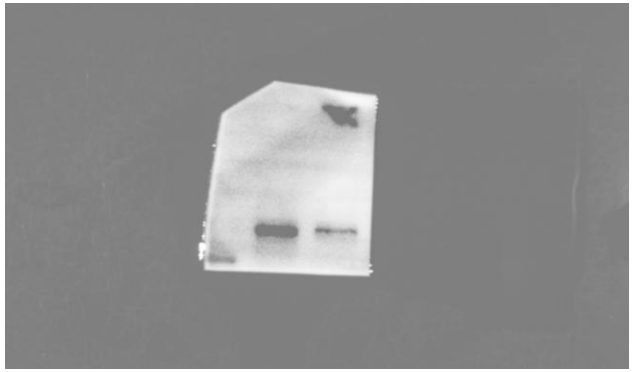

GAPDH

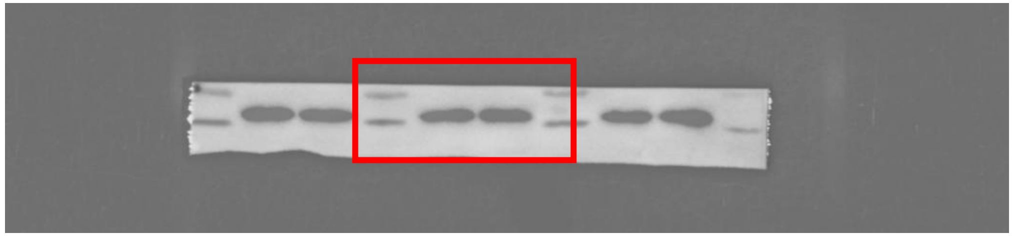

Full unedited gel for Figure 7C——Hs578T

PI3K

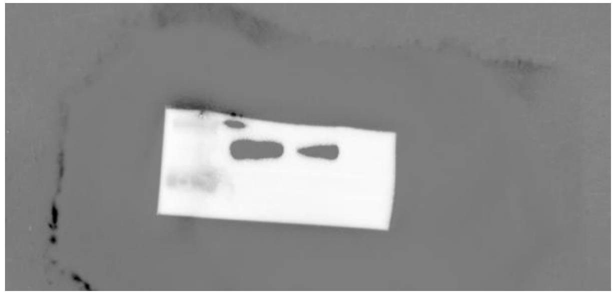

AKT

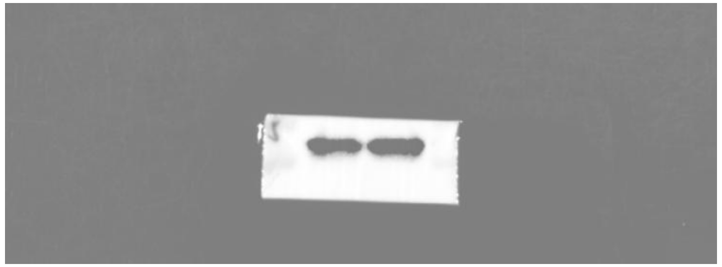

mTOR

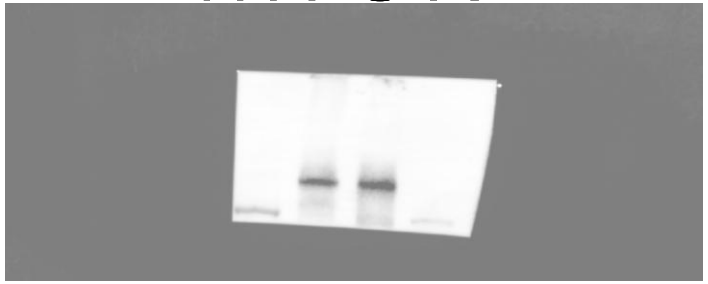

p-AKT

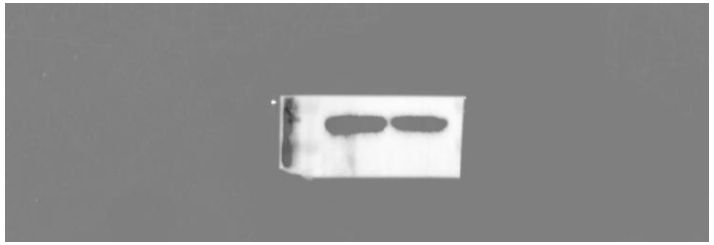

p-mTOR

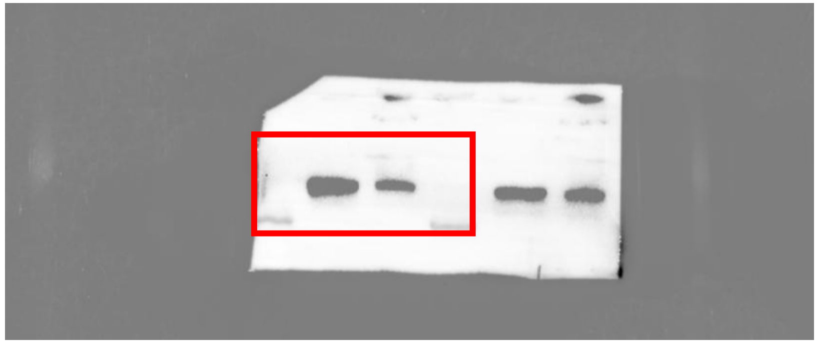

GAPDH

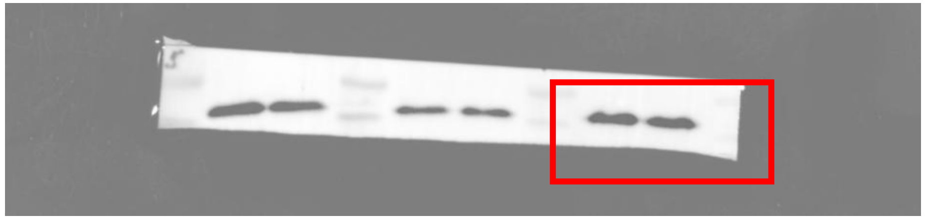

Supplement: Supplementary file 3 [file DataSheet1.pdf]
